# Racial/Ethnic Disparities in Risk of Breast Cancer Mortality by Molecular Subtype and Stage at Diagnosis

**DOI:** 10.1007/s10549-021-06311-7

**Published:** 2021-10-15

**Authors:** Nicole C. Lorona, Kathleen E. Malone, Christopher I. Li

**Affiliations:** 1. University of Washington, Seattle, WA, USA; 2. Fred Hutchinson Cancer Research Center, Seattle, WA, USA

**Keywords:** race, disparities, molecular subtype, mortality, breast cancer

## Abstract

**Purpose:**

Previous research has found significant survival disparities between Black and White women among select stages and subtypes of breast cancer, however other racial/ethnic groups have been less well-studied. This study expands on previous research, examining differences in breast cancer-specific mortality across multiple racial and ethnic groups.

**Methods:**

Women diagnosed with a first primary invasive breast cancer between 2010 and 2016 who were 20-85 years of age at diagnosis were identified from 18 Surveillance, Epidemiology, and End Results (SEER) registries. Subtypes were defined by joint hormone receptor (HR) and human epidermal growth factor receptor 2 (HER2) status. Cox proportional hazards models for each stage and subtype were fit, with non-Hispanic white women as the reference group. Effect modification by age at diagnosis (<50, ≥50) was found and thus analyses were age-stratified.

**Results:**

After multivariable adjustment, younger Black women had greater risks of breast cancer-specific death for all stages of HR+/HER2−, and certain stages of HR+/HER2+, TN, and HR−/HER2+ breast cancer. Asian/Pacific Islander women generally had a lower hazard of breast cancer-specific death. Older Hispanic White women had a lower hazard of breast cancer-specific death for stages I-III HR+/HER2− and stage II TN breast cancer.

**Conclusions:**

These findings demonstrate that different racial/ethnic groups experience different risks of breast cancer-specific mortality by stage and subtype. Efforts to address survival disparities should place additional focus on young Black women, as they experience meaningful disparities in breast cancer-specific mortality.

## Introduction

Joint estrogen receptor (ER) and progesterone receptor (PR), or hormone receptor (HR), status and human epidermal growth factor receptor 2 (HER2) overexpression serve as the basis of four well-established molecular subtypes of breast cancer: HR+/HER2−, HR+/HER2+, ER−/PR−/HER2−, or triple-negative (TN), and HR−/HER2+. HR+/HER2− breast cancers have the best prognosis, while TN cancers have the worst prognosis, in terms of 4-year breast cancer specific survival.[1,2] Additionally, while HR−/HER2+ tumors are generally more aggressive, they have targeted therapies available that can improve survival outcomes.[3]

Black, Hispanic White, Pacific Islander, and American Indian/Alaska Native women are 20-60% more likely than non-Hispanic White women to be diagnosed with advanced stage tumors,[4] and have 1.4 to 2.4 times increased higher risk of breast cancer-specific mortality,[5] with Black women in particular having significantly worse survival than women of other racial/ethnic groups.[6]

Previous population-based studies have found significant disparities between Black and White women in overall and breast cancer-specific mortality among select stages and subtypes of breast cancer, but not among all stages and subtypes.[7,8] Specifically, these studies found that Black women had worse cancer-specific survival[7] and a higher risk of cancer-specific mortality[8] in stage II and III of the HR+/HER2− subtype.[7,8] stage II of the HR+/HER2+ subtype,[7] stage IV of the HR−/HER2+ subtype,[7] and stages II[7] and III[8] of the TN subtype. These studies, however, focused exclusively on comparing Black women with White women, and did not include other racial/ethnic groups. Using the Surveillance, Epidemiology, and End Results (SEER) registries, which cover about 35% of the US population as a whole and have documented HER2 status since 2010 for breast cancer cases, the present study expands these findings by examining disparities in risk of breast cancer-specific mortality across multiple racial and ethnic groups. As women of different racial/ethnic backgrounds may have differences in tumor-related factors, experiences with the health care system, socioeconomic factors, and stress over the life-course that can also impact breast cancer-specific mortality [9], we hypothesized that the risk of breast cancer-specific mortality would be different for women of racial/ethnic minorities than for non-Hispanic White women across multiple subtypes and stages at diagnosis.

## Methods

### Study Population

The SEER program is comprised of 18 population-based cancer registries (Atlanta, Connecticut, Detroit, Hawaii, Iowa, New Mexico, San Francisco-Oakland, Seattle-Puget Sound, Utah, Los Angeles, San Jose-Monterey, Rural Georgia, Alaska Native Tumor Registry, Greater California, Greater Georgia, Kentucky, Louisiana, and New Jersey) that together cover 34.6% of the U.S. population, collecting information on all cancer cases diagnosed in their corresponding geographic regions.[10] In each of the SEER registries, patient information is extracted from medical records for each case and de-identified information is submitted to the SEER database.[11] SEER collects vital status from the National Center for Health Statistics and provides a cause-specific death classification using an algorithm that considers cause of death, tumor occurrence, original tumor site, and other comorbidities.[12] With the 2018 SEER data submission, follow-up for vital status is available through 12/31/16.[13] SEER has documented the status of two HRs, estrogen receptor (ER) and progesterone receptor (PR), since 1990 and has captured HER2 receptor status beginning in 2010. SEER defines, and provides a variable for, four breast cancer subtypes by joint HR and HER2 status: HR+/HER2−, HR+/HER2+, HR−/HER2− (TN), and HR−/HER2+[14] These data are publicly accessible after the submission of a signed data use agreement to the SEER program, with treatment data also publicly accessible after submission of an additional data use agreement. Data on surgery type, chemotherapy, and radiation therapy are available, but data on hormonal treatment and other targeted therapies are not included in current SEER data releases.

In this study, de-identified data on patient demographics, cancer characteristics, treatment, outcomes as of 12/31/2016, and survival time in months was collected from the SEER database[13] using SEER*Stat software[15] for women diagnosed with a first primary invasive breast cancer between 2010 and 2016 who were 20-85 years of age at diagnosis. A total of 343,499 cases meeting inclusion criteria were identified. Cases with missing follow-up information, unknown race, unknown molecular subtype, unknown stage, stage 0, or stage NOS were excluded (n=34,474). Cases with <1 month of follow-up information (n=5,506), missing data on definitive local treatment(n=8,936), missing data on insurance status (n=4,141), or missing cause of death (n=397) were also excluded from analyses resulting in a final analytic set of 290,045.

### Race/ethnicity

Race/ethnicity was divided into five mutually exclusive groups: Non-Hispanic White, Black, Hispanic White, Asian/Pacific Islander, and American Indian/Alaskan Native. Less than two percent of Black women in this sample were also classified as Hispanic (n = 398) so non-Hispanic Black and Hispanic Black women were combined in the same group. Detailed Hispanic subgroups, restricted to Hispanic White women, included Cuban, Dominican, Mexican, Puerto Rican, South/Central American, and other specified Hispanic origin. Data on Hispanic ethnic subgroups were only available for cases diagnosed between 2010 and 2015, with follow-up through 12/31/2015, so analyses for Hispanic ethnic subgroups are restricted to those years. Detailed Asian/Pacific Islander subgroups are available from all registries and include Korean, Chinese, Japanese, Filipino, Pacific Islander, Southeast Asian, Indian Subcontinent, and other specified Asian ethnicity.

### Statistical analysis

Unadjusted Kaplan-Meier survival curves were used to summarize differences in survival between racial/ethnic groups for each subtype and a stratified log-rank test was used to compare curves within strata of stage. Multivariable-adjusted Cox proportional hazards models were fit to estimate hazard ratios (HRs) and their associated 95% confidence intervals (CIs) for the association between race/ethnicity and breast cancer-specific mortality, with Non-Hispanic White women serving as the reference group in all models. Separate models were fit for each stratum of subtype and stage. Cases who remained alive were censored at their month of last known follow-up. Models for Hispanic White subgroups excluded women who were flagged as Hispanic by surname match alone or who were Hispanic NOS (n=410).

Effect modification by age at diagnosis and insurance status (uninsured/any Medicaid, insured/insured – no specifics) was assessed through likelihood ratio testing, Four different models were fit to adjust for different sets of confounders, adding in additional covariates with each model. Model 1 adjusted for age at diagnosis (as a continuous variable) and year of diagnosis (as a categorical variable). Model 2 adjusted for the covariates included in Model 1, and tumor grade (1, 2, 3/4, unknown). Model 3 adjusted for the covariates included in Model 2 and receipt of chemotherapy (yes, no/unknown) and definitive local treatment (breast-conserving surgery with radiation, total mastectomy with or without radiation, other). Model 4 adjusted for the covariates included in Model 3 and insurance status (uninsured, any Medicaid, insured). The county-level proportion of individuals living below the poverty level in 2010 was examined as a potential confounder but it did not change estimates by more than 10% and was not included in the final models.

The proportional hazards assumption was assessed by testing the correlation of the scaled Schoenfeld residuals and ranked failure time and by examining log-log Cox adjusted survival curves, and no strong evidence of violation of the assumption was found. Cells with fewer than five breast cancer-specific deaths are not shown. P-values less than 0.05 were considered significant, and all hypothesis tests were two-sided. All analyses were completed using Stata SE 15.0 software.

## Results

In this sample, non-Hispanic White women were on average older and Hispanic White women were on average younger (means = 60.8 and 55.7 years, respectively) compared to women of other racial/ethnic groups (Table 1). Among non-Hispanic White women, the proportions diagnosed with HR+/HER2− disease, diagnosed at earlier stages, diagnosed with lower grade disease, not treated with chemotherapy (or with unknown chemotherapy status), treated with breast-conserving surgery with radiation, and insured were all greater than the equivalent proportions among other groups. Black women were more likely to be diagnosed with TN breast cancer, to be diagnosed at later stages, to be diagnosed with grade III or IV disease, to not have received definitive local treatment, and to reside in counties where larger proportions of the population have household incomes below the poverty level. Hispanic White women were less likely than Black women, but more likely than non-Hispanic White women, to be diagnosed at later stages or with grade III or IV disease and were least likely to have had breast-conserving surgery with radiation. Asian-American women were least likely to be diagnosed with TN breast cancer, to be diagnosed with stage IV disease, and to live where larger proportions of the population have household incomes below the poverty level.

**Figure 1.**
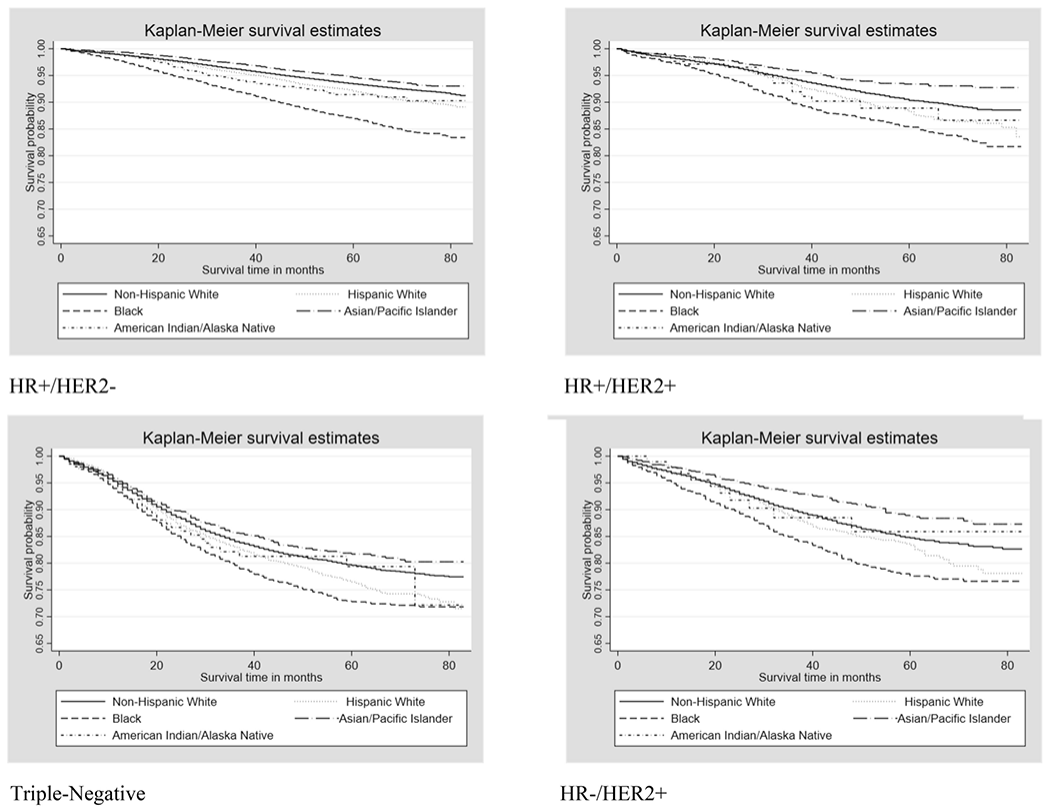
Kaplan-Meier curves for breast cancer-specific survival by molecular subtype among Non-Hispanic White, Hispanic White, Black, Asian/Pacific Islander, and American Indian/Alaska Native women diagnosed with breast cancer between 2010-2016, SEER 18 registries. P-value<0.001 for all stratified log-rank tests, with stage as stratifying variable.

There were 26,685 deaths from any cause, 17,910 of which were attributed to breast cancer, between January 1^st^, 2010 and December 31^st^, 2016. There was a median follow-up time of 34 months and a maximum follow-up time of 83 months. Unadjusted Kaplan-Meier survival curves for women of all stages combined for each subtype are presented in Figure 1. In each subtype, Black women had poorer breast cancer-specific survival than other racial/ethnic groups, and Asian/Pacific Islander women experienced better breast cancer-specific survival. In each subtype, the probability of breast cancer-specific mortality differed between women of different racial/ethnic groups within strata of stage (stratified log-rank test p-values <0.001). Age at diagnosis (<50 years, 50+ years) was a statistically significant effect modifier for over half of the stage and subtype combinations in each racial/ethnic group, but there was no effect modification by insurance status. Models additionally including an interaction term for binary age at diagnosis, with a linear age term for the main effect, were fit. Results for all subtypes are shown in Table 2, all stratified by age and adjusted for age at diagnosis, year at diagnosis, tumor grade, definitive local treatment, chemotherapy, and insurance status. Results for all models fit are reported in Supplementary Tables 1–3. Data is not shown for American Indian/Alaska Native cases due to there being less than 5 deaths for most of the subtypes and stages in each age group.

Black women younger than 50 at diagnosis had an over 50% greater risk of breast cancer-specific death across all stages of HR+/HER2− and stages II-IV HR+/HER2+ disease (data not shown for stage I HR+/HER2+ because less than 5 breast cancer deaths occurred in this group) compared to non-Hispanic White women. This observed disparity persisted after adjustment for grade, treatment characteristics, and insurance status (Supplementary Table 1). For TN and HR−/HER2+ disease, young Black women had slightly elevated risks in some stages that were mostly explained by treatment and insurance status, except for stage I HR−/HER2+ where a four times greater hazard of breast cancer-specific death was estimated after adjustment for age, year, grade, treatment, and insurance status (HR: 4.09; 95% CI: 1.50, 11.15). Black women aged 50 or older at diagnosis had an over 50% greater risk of breast cancer-specific mortality for stage IV HR+/HER2− and stage III-IV HER+/HER2+ breast cancer but did not have meaningful differences in risks for TN and HR−/HER2+ disease, compared to non-Hispanic White women of the same age group.

For stages I-III HR+/HER2− and HR+/HER2+ breast cancer, Asian/Pacific Islander women generally had a lower hazard of breast cancer-specific mortality than non-Hispanic White women, and this lower hazard was more pronounced among older women for HR+/HER2− breast cancer. Older Asian/Pacific Islander women with TN or HR−/HER2+ breast cancer also generally had a lower risk of breast cancer-specific death, while younger women had similar risk of mortality compared to non-Hispanic Whites. For HR+/HER2− and stages III/IV TN disease, results did not differ greatly between Chinese, Filipino, and Indian Subcontinent ethnic groups (Table 3). For early stage TN breast cancer, older Chinese and Filipino women had significantly lower risks of mortality than non-Hispanic white women, while Indian Subcontinent women had similar risks to non-Hispanic White women. Results for HR−/HER2+ or HR+/HER2+ disease by Asian/Pacific Islander ethnic subgroup could not be presented due to small sample size.

Compared to non-Hispanic White women, Hispanic White women aged 50 or older at diagnosis with stages I-III HR+/HER2− disease had 20-40% lower risks of breast cancer-specific death, while younger Hispanic White women had higher risks of breast cancer-specific death for later stage HR+/HER2− disease that were not statistically significant after adjustment for treatment and insurance status, respectively (Supplementary Table 3). Hispanic White women with TN and HR−/HER2+ breast cancer had similar risks of death compared to non-Hispanic White women in both age groups, except that older women with stage II TN breast cancer had a lower risk of breast cancer-specific mortality. Heterogeneity in breast cancer-specific survival was observed among Mexican, Puerto Rican, and South/Central American ethnic groups (Table 4). Younger Puerto Rican women with early stage (I/II) TN breast cancer had the highest risks of breast cancer-specific mortality that persisted after accounting for tumor grade, treatment, and insurance status (HR: 2.94; 95% CI: 1.38, 6.27); however, the sample size was small (n=39). Similarly, younger South/Central American women with early stage HR+/HER2− disease had higher, and older women had lower, risks of breast cancer-specific mortality that was not fully explained by grade, treatment, or insurance status. Results for HR−/HER2+ or HR+/HER2+ disease by Hispanic ethnic subgroup could not be presented due to small sample size.

## Discussion

In this large population-based study, disparities in risk of breast cancer-specific mortality were observed across women of different races/ethnicities and by stage, tumor subtype, and age. Black women under 50 years old at diagnosis experienced disparities of the largest magnitude compared to non-Hispanic White women. In contrast, older Hispanic White and Asian/Pacific Islander women experienced lower risks of breast cancer specific mortality. The present study is the first, to our knowledge, to examine disparities in risk of mortality among more than two racial/ethnic groups, stage, and subtype while also stratifying by age at diagnosis. Other similar studies did not assess effect modification by age at diagnosis[7,8,16] although young age at breast cancer diagnosis has been associated with poorer prognosis,[17] particularly among HR+ subtypes,[18,19] and an interaction between race/ethnicity and linear age has been observed in a survival analysis adjusting for stage and subtype.[20] Two previous studies focused exclusively on comparing Black women with White women and did not include other racial/ethnic groups, [7,8] while this study, along with others found that other racial/ethnic groups also experience different survival than non-Hispanic White women.[16,21] Additionally, Arciero et al. did not separate Hispanic White women from non-Hispanic White women in their reference group,[7] and Hispanic White women in the present study experienced different risks of death from breast cancer than non-Hispanic White women. The overall disparities among Black women and the lower risk of breast cancer-specific mortality observed among Asian/Pacific Islander women are consistent with previous literature.[5–8,16,21] Additionally, results from previous studies on Hispanic disparities in breast cancer-specific mortality are conflicting, and differences in findings may be explained by unexplored effect modification by age in these studies.[5,6,16,21]

By considering different models adjusting for different factors we were able to observe whether adjusting for certain variables accounted for initially observed disparities. Adjusting for tumor grade reduced the disparity for Black women mainly for the HR+/HER2− subtype, and to a lesser degree for other subtypes. Generally, a greater proportion of observed disparities were explained by insurance status, so insurance status, as a proxy for other health care and social factors, may drive some of the observed differences in mortality. Factors such as access to and quality of health care, stress over the life course, racism, and health behaviors are examples of factors related to health care and social factors that could contribute to risk of breast cancer-specific mortality[9]. Many of the observed disparities were still statistically significant after adjustment for tumor grade, treatment characteristics, and insurance status, indicating that other factors, such as hormonal treatment or obesity, may be responsible for the observed disparities. Some prior studies have shown that Black and Hispanic women are less likely to receive hormonal treatment than non-Hispanic white women,[22,23] but one study restricted to postmenopausal women did not find differences in hormonal therapy use by race or ethnicity. [24] Hormonal treatment is associated with a lower risk of breast cancer-specific death, and if young Black women in this study were less likely to receive hormonal treatment than non-Hispanic White women, then differences in hormonal treatment may explain the remaining survival disparity in HR+ disease. Moreover, obesity and diabetes are associated with breast cancer-specific mortality and may differ between racial/ethnic groups, which could contribute to the disparities that remained after adjustment for measured confounders.[25–27] In addition to obesity and diabetes, other potential explanations for the reduced risk of breast cancer-specific mortality observed among Asian/Pacific Islander women relative to non-Hispanic White women in this study include differences in diet [28] and physical activity [29].

One limitation of this study is the completeness of radiation therapy and chemotherapy as adjustment variables.[30] For cases missing information on whether radiation or chemotherapy was received, it is unclear whether they did not receive these treatments or whether the registry failed to capture this data, which prevented sub-analyses stratifying by treatment.[30] Furthermore, we did not have detailed radiation or chemotherapy information, beyond whether the patient received it or not, or information on targeted or hormone therapies. This may result in residual confounding by treatment factors, if women of different racial/ethnic groups were more, or less, likely to receive these treatments than non-Hispanic white women. The present study, like other registry-based studies, is also limited by the lack of data on hormonal treatment and targeted therapies, other important comorbidities, lifestyle factors, individual and health systems-level social determinants of health (e.g., education, income, acculturation, occupation, health care provider details), and reproductive history that may confound the relationship between race/ethnicity and mortality. Although registry data may fail to capture these important variables, the observed disparities in this study warrant additional attention, regardless of their cause. We also excluded 15% of the initial population due to missing data, which may have introduced bias into our results if the data were not missing completely at random. Finally, this study uses broad racial/ethnic categories and heterogeneity within subgroups exists; however, small sample sizes of ethnic groups combined with multiple strata and short follow-up prevents analysis of more detailed ethnic groups beyond what was examined.

To summarize, these findings demonstrate that women of different racial/ethnic groups experience different breast cancer-specific mortality than non-Hispanic White women in certain stages and subtypes, with age at diagnosis acting as an important effect modifier. Further research of protective factors that may contribute to the reduced risk of breast cancer-specific mortality of Asian/Pacific Islander and Hispanic White women of certain subtypes is warranted. Additionally, future studies could examine whether other tumor characteristics may mediate these relationships. Efforts to address disparities in breast cancer-specific mortality should also place additional focus on young Black women, as they bear a disproportionate breast cancer mortality burden compared to non-Hispanic White women, particularly for HR+ subtypes.

## Supplementary Material

1756017-supmaterial

## Figures and Tables

**Table 1.** Distribution of demographic and clinical characteristics across racial/ethnic groups among women diagnosed with breast cancer between 2010-2016, SEER 18 registries.

Variable	Non-Hispanic White (n=196,134) n, %	Black (n=32,911) n, %	Hispanic White (n=32,398) n, %	Asian/Pacific Islander (n=26,791) n, %	American Indian/Alaska Native (n=1,811) n, %
**Mean age at breast cancer diagnosis (standard deviation)**	60.8 (12.3)	57.6 (12.6)	55.7 (12.7)	56.7 (12.4)	57.5 (12.0)
**Age group**					
<50	38,372 (19.6)	8,939 (27.2)	11,123 (34.3)	8,347 (31.2)	478 (26.4)
50-65	77,991 (39.8)	14,007 (42.6)	12,763 (39.4)	10,954 (40.9)	792 (43.7)
65+	79,771 (40.7)	9,965 (30.3)	8,512 (26.3)	7,490 (28.0)	541 (29.9)
**Year of breast cancer diagnosis**					
2010	26,807 (13.7)	4,228 (12.9)	3,854 (11.9)	3,311 (12.4)	221 (12.2)
2011	27,941 (14.3)	4,557 (13.9)	4,465 (13.8)	3,546 (13.2)	257 (14.2)
2012	28,076 (14.3)	4,746 (14.4)	4,561 (14.1)	3,630 (13.6)	258 (14.3)
2013	28,440 (14.5)	4,762 (14.5)	4,730 (14.6)	3,872 (14.5)	275 (15.2)
2014	28,739 (14.7)	4,876 (14.8)	4,716 (14.6)	4,024 (15.0)	280 (15.5)
2015	29,323 (15.0)	5,087 (15.5)	5,145 (15.9)	4,312 (16.1)	264 (14.6)
2016	26,808 (13.7)	4,655 (14.1)	4,927 (15.2)	4,096 (15.3)	256 (14.1)
**Subtype**					
HR+/HER2−	148,745 (75.8)	20,152 (61.2)	22,470 (69.4)	19,329 (72.2)	1,263 (69.7)
HR+/HER2+	20,319 (10.4)	3,928 (11.9)	4,098 (12.7)	3,397 (12.7)	234 (12.9)
TN	19,088 (9.7)	6,847 (20.8)	3,974 (12.3)	2,312 (8.6)	208 (11.5)
HR−/HER2+	7,982 (4.1)	1,984 (6.0)	1,856 (5.7)	1,753 (6.5)	106 (5.9)
**Stage**					
I	104,140 (53.1)	13,072 (39.7)	13,711 (42.3)	12,802 (47.8)	821 (45.3)
II	62,498 (31.9)	12,433 (37.8)	12,234 (37.8)	9,880 (36.9)	664 (36.7)
III	20,230 (10.3)	4,966 (15.1)	4,848 (15.0)	2,992 (11.2)	228 (12.6)
IV	9,266 (4.7)	2,440 (7.4)	1,605 (5.0)	1,117 (4.2)	98 (5.4)
**Grade**					
I	48,034 (24.5)	4,582 (13.9)	5,967 (18.4)	5,282 (19.7)	387 (21.4)
II	85,509 (43.6)	11,951 (36.3)	13,467 (41.6)	11,634 (43.4)	719 (39.7)
IIMV	55,434 (28.3)	14,699 (44.7)	11,747 (36.3)	8,862 (33.1)	624 (34.5)
Unknown	7,157 (3.7)	1,679 (5.1)	1,217 (3.8)	1,013 (3.8)	81 (4.5)
**Chemotherapy**					
No/Unknown	117,410 (59.9)	14,734 (44.8)	15,966 (49.3)	14,603 (54.5)	898 (49.6)
Yes	78,724 (40.1)	18,177 (55.2)	16,432 (50.7)	12,188 (45.5)	913 (50.4)
**Definitive local treatment**					
Breast-conserving surgery and radiation	92,557 (47.2)	13,411 (40.8)	11,965 (36.9)	10,645 (39.7)	750 (41.4)
Total mastectomy with or without radiation	72,034 (36.7)	13,056 (39.7)	13,993 (43.2)	11,868 (44.3)	785 (43.4)
Other	31,543 (16.1)	6,444 (19.6)	6,440 (19.9)	4,278 (16.0)	276 (15.2)
**Insurance status**					
Uninsured	2,155 (1.1)	1,048 (3.2)	1,210 (3.7)	444 (1.7)	23 (1.3)
Any Medicaid	15,227 (7.8)	6,725 (20.4)	8,729 (26.9)	4,033 (15.1)	639 (35.3)
Insured	178,752 (91.1)	25,138 (76.4)	22,459 (69.3)	22,314 (83.3)	1,149 (63.5)
**Proportion of persons in county below poverty level by quartile, 2010**					
≤11.51%	54,237 (27.7)	4,165 (12.7)	5,192 (16.0)	9,524 (35.6)	602 (33.2)
11.52% - 14.69%	57,309 (29.2)	6,280 (19.1)	7,397 (22.8)	8,265 (30.9)	374 (20.7)
14.70% - 18.39%	44,777 (22.8)	9,448 (28.7)	12,410 (38.3)	6,796 (25.4)	289 (15.9)
≥18.40%	39,798 (20.3)	13,018 (39.6)	7,397 (22.8)	2,206 (8.2)	546 (30.2)
Missing	13	0	2	0	0
**SEER registry**					
Alaska Native Tumor Registry	0	0	0	0	368 (20.3)
Atlanta	6,019 (3.1)	4,483 (13.6)	505 (1.6)	503 (1.9)	16 (0.9)
Greater California	42,021 (21.4)	2,921 (8.9)	12,614 (38.9)	6,147 (22.9)	472 (26.1)
Connecticut	11,248 (5.7)	1,117 (3.4)	994 (3.1)	323 (1.2)	20 (1.1)
Detroit	10,749 (5.5)	3,621 (11.0)	327 (1.0)	411 (1.5)	41 (2.3)
Greater Georgia	15,372 (7.8)	5,246 (15.9)	503 (1.6)	268 (1.0)	29 (1.6)
Hawaii	1,242 (0.6)	56 (0.2)	142 (0.4)	3,967 (14.8)	22 (1.2)
Iowa	10,586 (5.4)	189 (0.6)	163 (0.5)	110 (0.4)	24 (1.3)
Kentucky	15,019 (7.7)	1,109 (3.4)	99 (0.3)	111 (0.4)	11 (0.6)
Los Angeles	12,231 (6.2)	2,872 (8.7)	7,492 (23.1)	4,731 (17.7)	59 (3.3)
Louisiana	10,522 (5.4)	4,959 (15.1)	305 (0.9)	155 (0.6)	25 (1.4)
New Jersey	22,304 (11.4)	3,804 (11.6)	2,817 (8.7)	2,007 (7.5)	36 (2.0)
New Mexico	3,281 (1.7)	86 (0.3)	1,979 (6.1)	69 (0.3)	305 (16.8)
Rural Georgia	350 (0.2)	154 (0.5)	3 (0.0)	7 (0.0)	0
San Francisco/Oakland	9,129 (4.7)	1,415 (4.3)	1,742 (5.4)	4,229 (15.8)	57 (3.2)
San Jose/Monterey	4,748 (2.4)	203 (0.6)	1,497 (4.6)	1,915 (7.2)	25 (1.4)
Seattle (Puget Sound)	15,006 (7.7)	642 (2.0)	686 (2.1)	1,643 (6.1)	255 (14.1)
Utah	6,307 (3.2)	34 (0.1)	530 (1.6)	195 (0.7)	46 (2.5)

**Table 2.** Hazard ratios and 95% confidence intervals for breast cancer-specific mortality comparing Hispanic White, Black, and Asian/Pacific Islander women to non-Hispanic White women diagnosed with four molecular subtypes breast cancer between 2010-2016, stratified by age at diagnosis, subtype and stage, SEER 18 registries.

	Black		Hispanic White		Asian/Pacific Islander	
	Age <50 (N=8,939)	Age 50+ (N=23,972)	Age <50 (N=11,123)	Age 50+ (N=21,275)	Age <50 (N=8,347)	Age 50+ (N=18,444)
**HR+/HER2−**	n=1,749	n=7,498	n=2,559	n=8,259	n=2,594	n=7,612
Stage I	1.88 (1.15, 3.07)*	0.98 (0.69, 1.40)	0.98 (0.56, 1.72)	0.61 (0.41, 0.89)*	0.56 (0.26, 1.21)	0.39 (0.25, 0.61)*
p for interaction	<0.001		<0.001		<0.001	

	n=2,004	n=5,027	n=2,849	n=5,033	n=2,235	n=4,386
Stage II	1.57 (1.24, 1.98)*	1.07 (0.88, 1.32)	0.92 (0.71, 1.20)	0.75 (0.60, 0.93)*	0.61 (0.43, 0.88)*	0.45 (0.35, 0.59)*
p for interaction	<0.001		<0.001		<0.001	

	n=813	n=1,794	n=1,204	n=1,674	n=712	n=1,137
Stage III	1.76 (1.45, 2.15)*	1.19 (0.99, 1.44)	1.11 (0.90, 1.38)	0.79 (0.64, 0.98)*	0.77 (0.57, 1.04)	0.64 (0.50, 0.81)*
p for interaction	<0.001		<0.001		<0.001	

	n=326	n=941	n=294	n=598	n=186	n=467
Stage IV	1.64 (1.36, 1.98)*	1.56 (1.33, 1.83)*	1.17 (0.95, 1.43)	1.10 (0.92, 1.31)	1.21 (0.94, 1.55)	1.04 (0.85, 1.26)
p for interaction	0.045		0.021		0.021	

**HR+/HER2+**	n=369	n=935	n=443	n=866	n=397	n=829
Stage I	†	0.87 (0.37, 2.07)	†	0.79 (0.32, 1.91)	†	†
p for interaction	0.179		0.482		0.204	

	n=564	n=979	n=794	n=924	n=602	n=854
Stage II	1.92 (1.19, 3.10)*	0.70 (0.42, 1.18)	0.65 (0.35, 1.23)	0.85 (0.51, 1.40)	0.37 (0.15, 0.92)*	0.47 (0.26, 0.86)*
p for interaction	<0.001		0.522		0.318	

	n=250	n=414	n=349	n=413	n=212	n=299
Stage III	2.05 (1.35, 3.10)*	1.93 (1.29, 2.89)*	1.27 (0.81, 2.01)	1.34 (0.87, 2.04)	0.77 (0.39, 1.50)	0.83 (0.49, 1.42)
p for interaction	0.143		0.818		0.580	

	n=144	n=273	n=126	n=183	n=72	n=132
Stage IV	1.71 (1.20, 2.42)*	1.52 (1.12, 2.07)*	1.03 (0.69, 1.54)	1.14 (0.81, 1.62)	1.49 (0.88, 2.51)	1.32 (0.90, 1.92)
p for interaction	0.438		0.647		0.535	

**Triple-Negative**	n=412	n=1,591	n=363	n=722	n=209	n=559
Stage I	1.04 (0.60, 1.78)	0.93 (0.60, 1.42)	0.70 (0.36, 1.33)	0.79 (0.47, 1.33)	0.98 (0.45, 2.16)	0.50 (0.26, 0.94)*
p for interaction	0.019		0.013		0.034	

	n=1,063	n=2,030	n=968	n=946	n=420	n=677
Stage II	1.21 (0.97, 1.50)	0.84 (0.67, 1.04)	0.84 (0.65, 1.08)	0.65 (0.50, 0.85)*	0.88 (0.62, 1.26)	0.46 (0.33, 0.65)*
p for interaction	<0.001		0.004		<0.001	

	n=468	n=774	n=373	n=389	n=128	n=199
Stage III	1.24 (1.01, 1.52)*	1.13 (0.91, 1.39)	1.09 (0.87, 1.36)	0.86 (0.67, 1.11)	1.05 (0.75, 1.48)	1.12 (0.82, 1.52)
p for interaction	0.265		0.111		0.520	

	n=142	n=367	n=90	n=123	n=39	n=81
Stage IV	0.88 (0.68, 1.14)	1.05 (0.83, 1.34)	1.07 (0.80, 1.43)	1.04 (0.77, 1.40)	1.00 (0.66, 1.51)	0.93 (0.66, 1.30)
p for interaction	0.593		0.569		0.717	

**HR−/HER2+**	n=136	n=382	n=160	n=339	n=170	n=432
Stage I	4.09 (1.50, 11.15)*	1.17 (0.39, 3.46)	†	0.60 (0.17, 2.14)	†	†
p for interaction	0.028		0.827		0.868	

	n=250	n=516	n=281	n=439	n=237	n=469
Stage II	1.51 (0.77, 2.99)	0.88 (0.49, 1.58)	1.08 (0.52, 2.22)	0.53 (0.28, 1.01)	†	†
p for interaction	0.105		0.014		0.033	

	n=167	n=286	n=190	n=256	n=95	n=210
Stage III	1.34 (0.85, 2.12)	0.93 (0.59, 1.46)	1.26 (0.79, 2.01)	0.86 (0.55, 1.36)	1.16 (0.64, 2.08)	0.59 (0.36, 0.98)*
p for interaction	0.183		0.504		0.269	

	n=82	n=165	n=80	n=111	n=39	n=101
Stage IV	1.52 (1.00, 2.31)*	1.05 (0.70, 1.57)	1.29 (0.83, 2.02)	0.96 (0.62, 1.50)	0.79 (0.40, 1.56)	0.75 (0.47, 1.21)
p for interaction	0.202		0.730		0.961	

* significant at p=0.05

a. Adjusted for age at diagnosis, year at diagnosis, tumor grade, definitive local treatment, chemotherapy, and insurance status (uninsured, any Medicaid, insured, insured/no specifics).

† <5 breast cancer deaths occurred in this group and thus HRs could not be reliably reported.

**Table 3.** Hazard ratios and 95% confidence intervals for breast cancer-specific mortality comparing different ethnic subgroups of Asian/Pacific Islander women to non-Hispanic White women diagnosed with HR+/HER2− or triple-negative breast cancer between 2010-2016, SEER 18 registries.

	Chinese		Filipino		Indian Subcontinent	

	Age <50 (N=1,189)	Age 50+ (N=2,553)	Age <50 (N=1,229)	Age 50+ (N=3,943)	Age<50 (N=847)	Age 50+ (N=1,412)
**HR+/HER2−**	n=914	n=2,040	n=904	n=3,073	n=545	n=1,064
Stage I/II ^a^	0.41 (0.17, 1.00)*	0.34 (0.21, 0.55)*	0.41 (0.17, 0.98)*	0.33 (0.23, 0.49)*	0.84 (0.37, 1.88)	0.48 (0.26, 0.86)*
p for interaction	<0.001		<0.001		<0.001	

	n=144	n=218	n=183	n=493	n=140	n=194
Stage III/IV	0.74 (0.46, 1.19)	0.79 (0.57, 1.10)	1.01 (0.69, 1.46)	0.76 (0.60, 0.97)*	0.91 (0.54, 1.51)	0.80 (0.56, 1.13)
p for interaction	0.904		0.359		0.839	

**Triple-Negative**	n=110	n=245	n=118	n=302	n=128	n=121
Stage I/II	1.50 (0.82, 2.75)	0.26 (0.12, 0.57)*	0.82 (0.40, 1.65)	0.48 (0.28, 0.85)*	0.82 (0.39, 1.74)	0.79 (0.42, 1.51)
p for interaction	<0.001		<0.001		<0.001	

	n=21	n=50	n=24	n=75	n=34	n=29
Stage III/IV	0.62 (0.27, 1.38)	1.36 (0.89, 2.08)	0.68 (0.32, 1.44)	1.21 (0.83, 1.75)	1.15 (0.68, 1.97)	0.48 (0.23, 1.04)
p for interaction	0.259		0.752		0.360	

* significant at p=0.05

a. Adjusted for age at diagnosis, year at diagnosis, tumor grade, definitive local treatment, chemotherapy, and insurance status (uninsured, any Medicaid, insured, insured/no specifics).

**Table 4.** Hazard ratios and 95% confidence intervals for breast cancer specific mortality comparing different ethnic subgroups of Hispanic White women to non-Hispanic White women diagnosed with HR+/HER− or triple-negative breast cancer between 2010-2015, SEER 18 registries

	Mexican		Puerto Rican		South/Central American	

	Age <50 (N=4,966)	Age 50+ (N=4,944)	Age <50 (N=623)	Age 50+ (N=633)	Age<50 (N=1,660)	Age 50+ (N=1,728)
**HR+/HER2−**	n=1,173	n=2,413	n=116	n=360	n=366	n=944
Stage I/II ^a^	1.43 (0.91, 2.23)	0.73 (0.51, 1.05)	†	0.65 (0.29, 1.49)	2.23 (1.18, 4.23)*	0.39 (0.20, 0.77)*
p for interaction	<0.001		<0.001		<0.001	

	n=424	n=505	n=32	n=70	n=81	n=180
Stage III/IV	1.10 (0.84, 1.43)	0.93 (0.73, 1.17)	1.22 (0.50, 2.94)	0.87 (0.53, 1.40)	1.11 (0.63, 1.97)	0.54 (0.35, 0.83)*
p for interaction	0.114		0.971		0.231	

**Triple-Negative**	n=316	n=319	n=39	n=45	n=73	n=118
Stage I/II	1.12 (0.74, 1.71)	0.93 (0.62, 1.40)	2.94 (1.38, 6.27)*	1.08 (0.44, 2.66)*	†	0.86 (0.46, 1.62)
p for interaction	<0.001		<0.001		<0.001	

	n=148	n=126	n=12	n=16	n=21	n=44
Stage III/IV	0.91 (0.68, 1.22)	0.61 (0.43, 0.88)*	1.52 (0.71, 3.24)	1.27 (0.64, 2.51)	0.77 (0.36, 1.64)	0.64 (0.35, 1.15)
p for interaction	0.092		0.899		0.947	

* significant at p=0.05

a. Adjusted for age at diagnosis, year at diagnosis, tumor grade, definitive local treatment, chemotherapy, and insurance status (uninsured, any Medicaid, insured, insured/no specifics).

† <5 breast cancer deaths occurred in this group and thus HRs could not be reliably reported.

## Data Availability

The datasets generated during and analyzed during the current study are publicly available from the Surveillance, Epidemiology, and End Results (SEER) program upon request, https://seer.cancer.gov/.
