# Supplementary material for: Racial/Ethnic Disparities in Risk of Breast Cancer Mortality by Molecular Subtype and Stage at Diagnosis
Source: Breast Cancer Res Treat. Author manuscript; Available in PMC 2022 Dec 1. (PMC8665898; doi:10.1007/s10549-021-06311-7)
Supplement: 1756017-supmaterial [file NIHMS1756017-supplement-1756017-supmaterial.docx]

Supplementary Table 1. Hazard ratios and 95% confidence intervals for breast cancer-specific mortality comparing Hispanic White, Black, and Asian/Pacific Islander women to non-Hispanic White women diagnosed with four molecular subtypes breast cancer between 2010-2016, stratified by age at diagnosis, subtype and stage, SEER 18 registries.

|  |  | **Black** | | **Hispanic White** | | **Asian/Pacific Islander** | |
| --- | --- | --- | --- | --- | --- | --- | --- |
|  | **Adjustment variables** | **Age <50**  **(N=8,939)** | **Age 50+**  **(N=23,972)** | **Age <50**  **(N=11,123)** | **Age 50+**  **(N=21,275)** | **Age <50**  **(N=8,347)** | **Age 50+**  **(N=18,444)** |
| **HR+/HER2-** | | n=1,749 | n=7,498 | n=2,559 | n=8,259 | n=2,594 | n=7,612 |
| Stage I | Age and year^a^ | 2.40 (1.48, 3.92)* | 1.02 (0.72, 1.45) | 1.25 (0.72, 2.19) | 0.59 (0.40, 0.86)* | 0.58 (0.26, 1.25) | 0.35 (0.23, 0.55)* |
|  | + tumor grade^b^ | 2.01 (1.24, 3.28)* | 0.93 (0.66, 1.32) | 1.19 (0.68, 2.08) | 0.58 (0.40, 0.85)* | 0.55 (0.25, 1.20) | 0.35 (0.23, 0.54)* |
|  | + treatments^c^ | 2.01 (1.23, 3.28)* | 1.09 (0.77, 1.55) | 1.10 (0.63, 1.92) | 0.68 (0.46, 0.99)* | 0.56 (0.26, 1.22) | 0.43 (0.28, 0.67)* |
|  | + insurance^d^ | 1.88 (1.15, 3.07)* | 0.98 (0.69, 1.40) | 0.98 (0.56, 1.72) | 0.61 (0.41, 0.89)* | 0.56 (0.26, 1.21) | 0.39 (0.25, 0.61)* |
|  | p for interaction | <0.001 | | <0.001 | | <0.001 | |
|  |  | n=2,004 | n=5,027 | n=2,849 | n=5,033 | n=2,235 | n=4,386 |
| Stage II | Age and year | 2.02 (1.60, 2.55)* | 1.31 (1.07, 1.60)* | 1.18 (0.91, 1.53) | 0.86 (0.69, 1.07) | 0.63 (0.44, 0.91)* | 0.49 (0.37, 0.64)* |
|  | + tumor grade | 1.72 (1.36, 2.16)* | 1.17 (0.95, 1.43) | 1.11 (0.85, 1.44) | 0.85 (0.68, 1.06) | 0.62 (0.43, 0.89)* | 0.47 (0.36, 0.62)* |
|  | + treatments | 1.70 (1.35, 2.15)* | 1.19 (0.97, 1.46) | 1.05 (0.81, 1.36) | 0.86 (0.69, 1.07) | 0.62 (0.43, 0.90)* | 0.49 (0.38, 0.64)* |
|  | + insurance | 1.57 (1.24, 1.98)* | 1.07 (0.88, 1.32) | 0.92 (0.71, 1.20) | 0.75 (0.60, 0.93)* | 0.61 (0.43, 0.88)* | 0.45 (0.35, 0.59)* |
|  | p for interaction | <0.001 | | <0.001 | | <0.001 | |
|  |  | n=813 | n=1,794 | n=1,204 | n=1,674 | n=712 | n=1,137 |
| Stage III | Age and year | 2.15 (1.77, 2.61)* | 1.31 (1.09, 1.58)* | 1.30 (1.05, 1.61)* | 0.86 (0.70, 1.05) | 0.84 (0.62, 1.13) | 0.70 (0.55, 0.89)* |
|  | + tumor grade | 1.91 (1.57, 2.33)* | 1.17 (0.97, 1.41) | 1.26 (1.01, 1.56)* | 0.80 (0.65, 0.99)* | 0.79 (0.58, 1.07) | 0.62 (0.49, 0.79)* |
|  | + treatments | 1.88 (1.55, 2.29)* | 1.29 (1.07, 1.55)* | 1.21 (0.97, 1.50) | 0.88 (0.72, 1.08) | 0.78 (0.57, 1.06) | 0.68 (0.53, 0.86)* |
|  | + insurance | 1.76 (1.45, 2.15)* | 1.19 (0.99, 1.44) | 1.11 (0.90, 1.38) | 0.79 (0.64, 0.98)* | 0.77 (0.57, 1.04) | 0.64 (0.50, 0.81)* |
|  | p for interaction | <0.001 | | <0.001 | | <0.001 | |
|  |  | n=326 | n=941 | n=294 | n=598 | n=186 | n=467 |
| Stage IV | Age and year | 1.82 (1.51, 2.19)* | 1.82 (1.56, 2.13)* | 1.29 (1.05, 1.58)* | 1.23 (1.03, 1.46)* | 1.30 (1.01, 1.66)* | 1.15 (0.95, 1.39) |
|  | + tumor grade | 1.74 (1.44, 2.10)* | 1.71 (1.46, 2.01)* | 1.25 (1.02, 1.54)* | 1.22 (1.02, 1.46)* | 1.25 (0.98, 1.61) | 1.10 (0.91, 1.34) |
|  | + treatments | 1.72 (1.42, 2.07)* | 1.67 (1.43, 1.95)* | 1.24 (1.01, 1.52)* | 1.19 (1.00, 1.42) | 1.23 (0.96, 1.58) | 1.12 (0.92, 1.36) |
|  | + insurance | 1.64 (1.36, 1.98)* | 1.56 (1.33, 1.83)* | 1.17 (0.95, 1.43) | 1.10 (0.92, 1.31) | 1.21 (0.94, 1.55) | 1.04 (0.85, 1.26) |
|  | p for interaction | 0.045 | | 0.021 | | 0.021 | |
| **HR+/HER2+** | | n=369 | n=935 | n=443 | n=866 | n=397 | n=829 |
| Stage I | Age and year | † | 0.94 (0.40, 2.19) | † | 0.91 (0.38, 2.20) | † | † |
|  | + tumor grade | † | 0.93 (0.40, 2.17) | † | 0.90 (0.37, 2.18) | † | † |
|  | + treatments | † | 1.08 (0.46, 2.54) | † | 1.01 (0.42, 2.42) | † | † |
|  | + insurance | † | 0.87 (0.37, 2.07) | † | 0.79 (0.32, 1.91) | † | † |
|  | p for interaction | 0.179 | | 0.482 | | 0.204 | |
|  |  | n=564 | n=979 | n=794 | n=924 | n=602 | n=854 |
| Stage II | Age and year | 2.19 (1.36, 3.52)* | 0.71 (0.42, 1.18) | 0.88 (0.47, 1.65) | 0.93 (0.57, 1.54) | 0.42 (0.17, 1.04) | 0.45 (0.25, 0.83)* |
|  | + tumor grade | 2.16 (1.35, 3.48)* | 0.70 (0.42, 1.18) | 0.87 (0.46, 1.64) | 0.94 (0.57, 1.56) | 0.41 (0.16, 1.03) | 0.45 (0.25, 0.82)* |
|  | + treatments | 2.20 (1.37, 3.53)* | 0.83 (0.49, 1.38) | 0.80 (0.43, 1.51) | 1.02 (0.62, 1.68) | 0.37 (0.15, 0.93)* | 0.55 (0.30, 0.99)* |
|  | + insurance | 1.92 (1.19, 3.10)* | 0.70 (0.42, 1.18) | 0.65 (0.35, 1.23) | 0.85 (0.51, 1.40) | 0.37 (0.15, 0.92)* | 0.47 (0.26, 0.86)* |
|  | p for interaction | <0.001 | | 0.522 | | 0.318 | |
|  |  | n=250 | n=414 | n=349 | n=413 | n=212 | n=299 |
| Stage III | Age and year | 2.33 (1.55, 3.53)* | 1.59 (1.06, 2.37)* | 1.54 (0.98, 2.42) | 1.41 (0.94, 2.14) | 0.79 (0.40, 1.54) | 0.77 (0.45, 1.31) |
|  | + tumor grade | 2.33 (1.54, 3.51)* | 1.57 (1.05, 2.34)* | 1.51 (0.96, 2.38) | 1.41 (0.94, 2.13) | 0.80 (0.41, 1.56) | 0.75 (0.45, 1.29) |
|  | + treatments | 2.22 (1.47, 3.36)* | 2.09 (1.40, 3.13)* | 1.47 (0.93, 2.31) | 1.59 (1.05, 2.39)* | 0.79 (0.40, 1.54) | 0.89 (0.52, 1.51) |
|  | + insurance | 2.05 (1.35, 3.10)* | 1.93 (1.29, 2.89)* | 1.27 (0.81, 2.01) | 1.34 (0.87, 2.04) | 0.77 (0.39, 1.50) | 0.83 (0.49, 1.42) |
|  | p for interaction | 0.143 | | 0.818 | | 0.580 | |
|  |  | n=144 | n=273 | n=126 | n=183 | n=72 | n=132 |
| Stage IV | Age and year | 1.70 (1.20, 2.41)* | 1.65 (1.21, 2.24)* | 1.22 (0.82, 1.81) | 1.20 (0.85, 1.70) | 1.45 (0.86, 2.45) | 1.35 (0.93, 1.96) |
|  | + tumor grade | 1.71 (1.21, 2.43)* | 1.63 (1.20, 2.22)* | 1.21 (0.82, 1.80) | 1.19 (0.84, 1.68) | 1.45 (0.86, 2.44) | 1.34 (0.92, 1.94) |
|  | + treatments | 1.87 (1.32, 2.65)* | 1.73 (1.28, 2.35)* | 1.24 (0.84, 1.85) | 1.34 (0.95, 1.89) | 1.49 (0.88, 2.51) | 1.50 (1.04, 2.19)* |
|  | + insurance | 1.71 (1.20, 2.42)* | 1.52 (1.12, 2.07)* | 1.03 (0.69, 1.54) | 1.14 (0.81, 1.62) | 1.49 (0.88, 2.51) | 1.32 (0.90, 1.92) |
|  | p for interaction | 0.438 | | 0.647 | | 0.535 | |
| **Triple-Negative** | | n=412 | n=1,591 | n=363 | n=722 | n=209 | n=559 |
| Stage I | Age and year | 1.04 (0.61, 1.80) | 0.89 (0.59, 1.36) | 0.79 (0.42, 1.51) | 0.80 (0.48, 1.33) | 0.97 (0.44, 2.12) | 0.47 (0.25, 0.89)* |
|  | + tumor grade | 1.02 (0.59, 1.75) | 0.86 (0.57, 1.32) | 0.76 (0.40, 1.45) | 0.78 (0.47, 1.31) | 0.98 (0.45, 2.15) | 0.48 (0.25, 0.91)* |
|  | + treatment | 1.05 (0.61, 1.80) | 0.95 (0.62, 1.45) | 0.73 (0.38, 1.39) | 0.82 (0.49, 1.37) | 0.97 (0.44, 2.13) | 0.50 (0.27, 0.96)* |
|  | + insurance | 1.04 (0.60, 1.78) | 0.93 (0.60, 1.42) | 0.70 (0.36, 1.33) | 0.79 (0.47, 1.33) | 0.98 (0.45, 2.16) | 0.50 (0.26, 0.94)* |
|  | p for interaction | 0.019 | | 0.013 | | 0.034 | |
|  |  | n=1,063 | n=2,030 | n=968 | n=946 | n=420 | n=677 |
| Stage II | Age and year | 1.29 (1.04, 1.60)* | 0.86 (0.70, 1.07) | 1.02 (0.79, 1.30) | 0.73 (0.56, 0.95)* | 0.91 (0.64, 1.29) | 0.46 (0.33, 0.64)* |
|  | + tumor grade | 1.29 (1.04, 1.59)* | 0.86 (0.69, 1.07) | 1.01 (0.79, 1.30) | 0.73 (0.56, 0.95)* | 0.91 (0.63, 1.29) | 0.47 (0.34, 0.66)* |
|  | + treatment | 1.32 (1.07, 1.64)* | 0.93 (0.75, 1.16) | 0.96 (0.75, 1.23) | 0.75 (0.57, 0.97)* | 0.90 (0.63, 1.29) | 0.50 (0.36, 0.70)* |
|  | + insurance | 1.21 (0.97, 1.50) | 0.84 (0.67, 1.04) | 0.84 (0.65, 1.08) | 0.65 (0.50, 0.85)* | 0.88 (0.62, 1.26) | 0.46 (0.33, 0.65)* |
|  | p for interaction | <0.001 | | 0.004 | | <0.001 | |
|  |  | n=468 | n=774 | n=373 | n=389 | n=128 | n=199 |
| Stage III | Age and year | 1.30 (1.07, 1.60)* | 1.07 (0.87, 1.32) | 1.20 (0.96, 1.51) | 0.87 (0.68, 1.12) | 1.08 (0.77, 1.52) | 1.07 (0.79, 1.45) |
|  | + tumor grade | 1.30 (1.06, 1.59)* | 1.08 (0.87, 1.33) | 1.20 (0.96, 1.50) | 0.87 (0.68, 1.13) | 1.09 (0.77, 1.53) | 1.08 (0.80, 1.47) |
|  | + treatment | 1.29 (1.06, 1.58)* | 1.17 (0.95, 1.45) | 1.15 (0.92, 1.44) | 0.91 (0.71, 1.17) | 1.07 (0.76, 1.52) | 1.16 (0.86, 1.58) |
|  | + insurance | 1.24 (1.01, 1.52)* | 1.13 (0.91, 1.39) | 1.09 (0.87, 1.36) | 0.86 (0.67, 1.11) | 1.05 (0.75, 1.48) | 1.12 (0.82, 1.52) |
|  | p for interaction | 0.265 | | 0.111 | | 0.520 | |
|  |  | n=142 | n=367 | n=90 | n=123 | n=39 | n=81 |
| Stage IV | Age and year | 0.95 (0.74, 1.23) | 1.06 (0.83, 1.34) | 1.14 (0.86, 1.52) | 1.00 (0.74, 1.35) | 1.03 (0.68, 1.56) | 0.93 (0.67, 1.31) |
|  | + tumor grade | 0.95 (0.74, 1.23) | 1.06 (0.83, 1.34) | 1.12 (0.84, 1.49) | 1.00 (0.74, 1.35) | 1.01 (0.67, 1.54) | 0.93 (0.67, 1.31) |
|  | + treatment | 0.93 (0.72, 1.20) | 1.13 (0.89, 1.43) | 1.16 (0.87, 1.55) | 1.08 (0.80, 1.46) | 1.01 (0.67, 1.54) | 0.96 (0.68, 1.35) |
|  | + insurance | 0.88 (0.68, 1.14) | 1.05 (0.83, 1.34) | 1.07 (0.80, 1.43) | 1.04 (0.77, 1.40) | 1.00 (0.66, 1.51) | 0.93 (0.66, 1.30) |
|  | p for interaction | 0.593 | | 0.569 | | 0.717 | |
| **HR-/HER2+** | | n=136 | n=382 | n=160 | n=339 | n=170 | n=432 |
| Stage I | Age and year | 5.26 (1.95, 14.14)* | 1.43 (0.48, 4.21) | † | 0.83 (0.24, 2.88) | † | † |
|  | + tumor grade | 4.99 (1.85, 13.46)* | 1.39 (0.47, 4.11) | † | 0.84 (0.24, 2.91) | † | † |
|  | + treatment | 4.72 (1.74, 12.76)* | 1.35 (0.46, 3.97) | † | 0.81 (0.23, 2.83) | † | † |
|  | + insurance | 4.09 (1.50, 11.15)* | 1.17 (0.39, 3.46) | † | 0.60 (0.17, 2.14) | † | † |
|  | p for interaction | 0.028 | | 0.827 | | 0.868 | |
|  |  | n=250 | n=516 | n=281 | n=439 | n=237 | n=469 |
| Stage II | Age and year | 1.85 (0.94, 3.63) | 0.93 (0.52, 1.66) | 1.33 (0.65, 2.72) | 0.63 (0.34, 1.19) | † | † |
|  | + tumor grade | 1.82 (0.93, 3.58) | 0.93 (0.52, 1.65) | 1.34 (0.65, 2.73) | 0.64 (0.34, 1.20) | † | † |
|  | + treatment | 1.70 (0.86, 3.34) | 0.96 (0.54, 1.71) | 1.21 (0.59, 2.47) | 0.62 (0.33, 1.16) | † | † |
|  | + insurance | 1.51 (0.77, 2.99) | 0.88 (0.49, 1.58) | 1.08 (0.52, 2.22) | 0.53 (0.28, 1.01) | † | † |
|  | p for interaction | 0.105 | | 0.014 | | 0.033 | |
|  |  | n=167 | n=286 | n=190 | n=256 | n=95 | n=210 |
| Stage III | Age and year | 1.31 (0.83, 2.06) | 0.95 (0.61, 1.48) | 1.30 (0.82, 2.06) | 0.95 (0.61, 1.49) | 1.08 (0.60, 1.95) | 0.62 (0.38,1.02) |
|  | + tumor grade | 1.31 (0.83, 2.06) | 0.95 (0.61, 1.49) | 1.30 (0.82, 2.06) | 0.96 (0.62, 1.51) | 1.09 (0.61, 1.97) | 0.63 (0.38, 1.03) |
|  | + treatment | 1.40 (0.89, 2.21) | 0.95 (0.60, 1.49) | 1.33 (0.83, 2.10) | 0.93 (0.60, 1.46) | 1.17 (0.65, 2.10) | 0.63 (0.38, 1.03) |
|  | + insurance | 1.34 (0.85, 2.12) | 0.93 (0.59, 1.46) | 1.26 (0.79, 2.01) | 0.86 (0.55, 1.36) | 1.16 (0.64, 2.08) | 0.59 (0.36, 0.98)* |
|  | p for interaction | 0.183 | | 0.504 | | 0.269 | |
|  |  | n=82 | n=165 | n=80 | n=111 | n=39 | n=101 |
| Stage IV | Age and year | 1.75 (1.16, 2.65)* | 1.14 (0.77, 1.70) | 1.41 (0.91, 2.20) | 1.14 (0.74, 1.77) | 0.84 (0.43, 1.64) | 0.71 (0.45, 1.14) |
|  | + tumor grade | 1.74 (1.15, 2.63)* | 1.16 (0.78, 1.72) | 1.38 (0.88, 2.15) | 1.18 (0.76, 1.82) | 0.86 (0.44, 1.70) | 0.72 (0.45, 1.15) |
|  | + treatment | 1.67 (1.11, 2.53)* | 1.16 (0.78, 1.73) | 1.42 (0.91, 2.21) | 1.12 (0.73, 1.74) | 0.79 (0.40, 1.55) | 0.84 (0.53, 1.34) |
|  | + insurance | 1.52 (1.00, 2.31)* | 1.05 (0.70, 1.57) | 1.29 (0.83, 2.02) | 0.96 (0.62, 1.50) | 0.79 (0.40, 1.56) | 0.75 (0.47, 1.21) |
|  | p for interaction | 0.202 | | 0.730 | | 0.961 | |

*significant at p=0.05

a. Adjusted for age at diagnosis (as a continuous variable) and year at diagnosis (as a categorical variable).

b. Adjusted for age at diagnosis, year at diagnosis, and tumor grade (I, II, III/IV , unknown).

c. Adjusted for age at diagnosis, year at diagnosis, tumor grade, and definitive local treatment (breast-conserving surgery and radiation, mastectomy with or without radiation, other) and chemotherapy (yes, no).

d. Adjusted for age at diagnosis, year at diagnosis, tumor grade, definitive local treatment, chemotherapy, and insurance status (uninsured, any Medicaid, insured, insured/no specifics).

† <5 breast cancer deaths occurred in this group and thus HRs could not be reliably reported.

Supplementary Table 2. Hazard ratios and 95% confidence intervals for breast cancer-specific mortality comparing different ethnic subgroups of Asian/Pacific Islander women to non-Hispanic White women diagnosed with HR+/HER2- or triple-negative breast cancer between 2010-2016, SEER 18 registries.

|  |  | **Chinese** | | **Filipino** | | **Indian Subcontinent** | |
| --- | --- | --- | --- | --- | --- | --- | --- |
|  | **Adjustment variables** | **Age <50**  **(N=1,189)** | **Age 50+**  **(N=2,553)** | **Age <50**  **(N=1,229)** | **Age 50+**  **(N=3,943)** | **Age<50**  **(N=847)** | **Age 50+**  **(N=1,412)** |
| **HR+/HER2-** |  | n=914 | n=2,040 | n=904 | n=3,073 | n=545 | n=1,064 |
| Stage I/II | Age and year^a^ | 0.42 (0.17, 1.01) | 0.34 (0.21, 0.56)* | 0.42 (0.18, 1.03) | 0.36 (0.25, 0.54)* | 0.93 (0.41, 2.08) | 0.48 (0.27, 0.87)* |
|  | + tumor grade^b^ | 0.40 (0.16, 0.96)* | 0.33 (0.21, 0.54)* | 0.42 (0.17, 1.00) | 0.35 (0.24, 0.51)* | 0.83 (0.37, 1.86) | 0.49 (0.27, 0.89)* |
|  | + treatment^c^ | 0.42 (0.17, 1.01) | 0.38 (0.24, 0.62)* | 0.41 (0.17, 0.98)* | 0.37 (0.25, 0.54)* | 0.85 (0.38, 1.90) | 0.56 (0.31, 1.00) |
|  | + insurance^d^ | 0.41 (0.17, 1.00)* | 0.34 (0.21, 0.55)* | 0.41 (0.17, 0.98)* | 0.33 (0.23, 0.49)* | 0.84 (0.37, 1.88) | 0.48 (0.26, 0.86)* |
|  | p for interaction | <0.001 | | <0.001 | | <0.001 | |
|  |  | n=144 | n=218 | n=183 | n=493 | n=140 | n=194 |
| Stage III/IV | Age and year | 0.81 (0.50, 1.30) | 0.81 (0.58, 1.12) | 1.14 (0.78, 1.65) | 0.84 (0.67, 1.07) | 0.83 (0.50, 1.38) | 0.95 (0.68, 1.34) |
|  | + tumor grade | 0.80 (0.50, 1.30) | 0.79 (0.57, 1.10) | 1.07 (0.74, 1.55) | 0.80 (0.63, 1.01) | 0.76 (0.46, 1.27) | 0.96 (0.68, 1.35) |
|  | + treatment | 0.75 (0.46, 1.21) | 0.84 (0.60, 1.17) | 1.05 (0.72, 1.52) | 0.83 (0.66, 1.05) | 0.91 (0.54, 1.52) | 0.89 (0.63, 1.26) |
|  | + insurance | 0.74 (0.46, 1.19) | 0.79 (0.57, 1.10) | 1.01 (0.69, 1.46) | 0.76 (0.60, 0.97)* | 0.91 (0.54, 1.51) | 0.80 (0.56, 1.13) |
|  | p for interaction | 0.904 | | 0.359 | | 0.839 | |
| **Triple-Negative** | | n=110 | n=245 | n=118 | n=302 | n=128 | n=121 |
| Stage I/II | Age and year | 1.51 (0.83, 2.75) | 0.25 (0.12, 0.54)* | 0.88 (0.43, 1.77) | 0.49 (0.28, 0.85)* | 0.81 (0.38, 1.71) | 0.80 (0.42, 1.51) |
|  | + tumor grade | 1.47 (0.81, 2.69) | 0.27 (0.13, 0.59)* | 0.89 (0.44, 1.79) | 0.52 (0.30, 0.91)* | 0.79 (0.38, 1.68) | 0.76 (0.40, 1.44) |
|  | + treatments | 1.49 (0.81, 2.71) | 0.28 (0.13, 0.60)* | 0.84 (0.41, 1.69) | 0.50 (0.29, 0.87)* | 0.80 (0.38, 1.70) | 0.83 (0.44, 1.59) |
|  | + insurance | 1.50 (0.82, 2.75) | 0.26 (0.12, 0.57)* | 0.82 (0.40, 1.65) | 0.48 (0.28, 0.85)* | 0.82 (0.39, 1.74) | 0.79 (0.42, 1.51) |
|  | p for interaction | <0.001 | | <0.001 | | <0.001 | |
|  |  | n=21 | n=50 | n=24 | n=75 | n=34 | n=29 |
| Stage III/IV | Age and year | 0.76 (0.34, 1.71) | 1.52 (1.00, 2.32) | 0.79 (0.37, 1.67) | 1.09 (0.75, 1.57) | 1.16 (0.68, 1.98) | 0.60 (0.28, 1.29) |
|  | + tumor grade | 0.76 (0.34, 1.70) | 1.57 (1.03, 2.39)* | 0.81 (0.38, 1.71) | 1.12 (0.78, 1.62) | 1.13 (0.66, 1.93) | 0.63 (0.30, 1.35) |
|  | + treatment | 0.64 (0.28, 1.43) | 1.45 (0.95, 2.21) | 0.70 (0.33, 1.48) | 1.28 (0.88, 1.84) | 1.16 (0.68, 1.98) | 0.53 (0.25, 1.13) |
|  | + insurance | 0.62 (0.27, 1.38) | 1.36 (0.89, 2.08) | 0.68 (0.32, 1.44) | 1.21 (0.83, 1.75) | 1.15 (0.68, 1.97) | 0.48 (0.23, 1.04) |
|  | p for interaction | 0.259 | | 0.752 | | 0.360 | |

*significant at p=0.05

a. Adjusted for age at diagnosis (as a continuous variable) and year at diagnosis (as a categorical variable).

b. Adjusted for age at diagnosis, year at diagnosis, and tumor grade (I, II, III/IV , unknown).

c. Adjusted for age at diagnosis, year at diagnosis, tumor grade, and definitive local treatment (breast-conserving surgery and radiation, mastectomy with or without radiation, other) and chemotherapy (yes, no).

d. Adjusted for age at diagnosis, year at diagnosis, tumor grade, definitive local treatment, chemotherapy, and insurance status (uninsured, any Medicaid, insured, insured/no specifics).

Supplementary Table 3. Hazard ratios and 95% confidence intervals for breast cancer specific mortality comparing different ethnic subgroups of Hispanic White women to non-Hispanic White women diagnosed with HR+/HER- or triple-negative breast cancer between 2010-2015, SEER 18 registries.

|  |  | **Mexican** | | **Puerto Rican** | | **South/Central American** | |
| --- | --- | --- | --- | --- | --- | --- | --- |
|  | **Adjustment variables** | **Age <50**  **(N=4,966)** | **Age 50+**  **(N=4,944)** | **Age <50**  **(N=623)** | **Age 50+**  **(N=633)** | **Age<50**  **(N=1,660)** | **Age 50+**  **(N=1,728)** |
| **HR+/HER2-** |  | n=1,173 | n=2,413 | n=116 | n=360 | n=366 | n=944 |
| Stage I/II | Age and year^a^ | 2.27 (1.46, 3.52)* | 0.88 (0.62, 1.27) | † | 0.77 (0.34, 1.75) | 3.28 (1.74, 6.20)* | 0.46 (0.24, 0.92)* |
|  | + tumor grade^b^ | 2.07 (1.33, 3.22)* | 0.89 (0.62, 1.28) | † | 0.75 (0.33, 1.70) | 2.94 (1.56, 5.54)* | 0.46 (0.23, 0.90)* |
|  | + treatment^c^ | 1.88 (1.21, 2.92)* | 0.92 (0.64, 1.33) | † | 0.81 (0.36, 1.85) | 2.71 (1.44, 5.12)* | 0.48 (0.24, 0.94)* |
|  | + insurance^d^ | 1.43 (0.91, 2.23) | 0.73 (0.51, 1.05) | † | 0.65 (0.29, 1.49) | 2.23 (1.18, 4.23)* | 0.39 (0.20, 0.77)* |
|  | p for interaction | <0.001 | | <0.001 | | <0.001 | |
|  |  | n=424 | n=505 | n=32 | n=70 | n=81 | n=180 |
| Stage III/IV | Age and year | 1.44 (1.11, 1.86)* | 1.03 (0.82, 1.30) | 1.34 (0.55, 3.23) | 1.23 (0.76, 1.98) | 1.22 (0.69, 2.16) | 0.65 (0.42, 1.01) |
|  | + tumor grade | 1.35 (1.04, 1.75)* | 1.01 (0.80, 1.28) | 1.20 (0.50, 2.89) | 1.14 (0.70, 1.84) | 1.17 (0.66, 2.07) | 0.64 (0.41, 0.99)* |
|  | + treatment | 1.28 (0.99, 1.66) | 1.09 (0.86, 1.38) | 1.34 (0.55, 3.23) | 1.02 (0.63, 1.65) | 1.22 (0.69, 2.16) | 0.63 (0.41, 0.97)* |
|  | + insurance | 1.10 (0.84, 1.43) | 0.93 (0.73, 1.17) | 1.22 (0.50, 2.94) | 0.87 (0.53, 1.40) | 1.11 (0.63, 1.97) | 0.54 (0.35, 0.83)* |
|  | p for interaction | 0.114 | | 0.971 | | 0.231 | |
| **Triple-Negative** | | n=316 | n=319 | n=39 | n=45 | n=73 | n=118 |
| Stage I/II | Age and year | 1.43 (0.95, 2.17) | 1.17 (0.79, 1.74) | 3.29 (1.55, 6.98)* | 1.23 (0.49, 2.99) | † | 1.03 (0.55, 1.92) |
|  | + tumor grade | 1.41 (0.93, 2.14) | 1.12 (0.75, 1.67) | 3.33 (1.57, 7.08)* | 1.13 (0.46, 2.78) | † | 1.01 (0.55, 1.90) |
|  | + treatment | 1.36 (0.90, 2.06) | 1.10 (0.74, 1.65) | 3.24 (1.52, 6.89)* | 1.18 (0.48, 2.92) | † | 1.01 (0.54, 1.89) |
|  | + insurance | 1.12 (0.74, 1.71) | 0.93 (0.62, 1.40) | 2.94 (1.38, 6.27)* | 1.08 (0.44, 2.66)* | † | 0.86 (0.46, 1.62) |
|  | p for interaction | <0.001 | | <0.001 | | <0.001 | |
|  |  | n=148 | n=126 | n=12 | n=16 | n=21 | n=44 |
| Stage III/IV | Age and year | 1.18 (0.89, 1.57) | 0.74 (0.52, 1.05) | 1.52 (0.72, 3.21) | 1.18 (0.59, 2.32) | 0.85 (0.40, 1.79) | 0.75 (0.42, 1.35) |
|  | + tumor grade | 1.18 (0.89, 1.58) | 0.74 (0.52, 1.05) | 1.40 (0.66, 2.96) | 1.16 (0.59, 2.30) | 0.87 (0.41, 1.84) | 0.76 (0.42, 1.36) |
|  | + treatment | 1.05 (0.79, 1.40) | 0.69 (0.49, 0.99)* | 1.79 (0.84, 3.79) | 1.42 (0.72, 2.81) | 0.91 (0.43, 1.94) | 0.79 (0.44, 1.42) |
|  | + insurance | 0.91 (0.68, 1.22) | 0.61 (0.43, 0.88)* | 1.52 (0.71, 3.24) | 1.27 (0.64, 2.51) | 0.77 (0.36, 1.64) | 0.64 (0.35, 1.15) |
|  | p for interaction | 0.092 | | 0.899 | | 0.947 | |

*significant at p=0.05

a. Adjusted for age at diagnosis (as a continuous variable) and year at diagnosis (as a categorical variable).

b. Adjusted for age at diagnosis, year at diagnosis, and tumor grade (I, II, III/IV , unknown).

c. Adjusted for age at diagnosis, year at diagnosis, tumor grade, and definitive local treatment (breast-conserving surgery and radiation, mastectomy with or without radiation, other) and chemotherapy (yes, no).

d. Adjusted for age at diagnosis, year at diagnosis, tumor grade, definitive local treatment, chemotherapy, and insurance status (uninsured, any Medicaid, insured, insured/no specifics).

† <5 breast cancer deaths occurred in this group and thus HRs could not be reliably reported.
